# *Ehrlichia ruminantium* infects *Rhipicephalus microplus* in West Africa

**DOI:** 10.1186/s13071-016-1651-x

**Published:** 2016-06-22

**Authors:** Abel Biguezoton, Valerie Noel, Safiou Adehan, Hassane Adakal, Guiguigbaza-Kossigan Dayo, Sébastien Zoungrana, Souaïbou Farougou, Christine Chevillon

**Affiliations:** Unité de Recherche en Biotechnologie de la Production et de la Santé Animales (URBPSA), Laboratoire de Recherche en Biologie Appliquée, Ecole Polytechnique d’Abomey-Calavi, 01 BP 2009, Cotonou, Bénin; Unité de Recherche sur les bases biologiques de la Lutte Intégrée (URBIO), Centre International de Recherche-Développement sur l’Elevage en zone Subhumide (CIRDES), 559, 3-51 Avenue du Gouverneur Louveau, 01B.P. 454, Bobo-Dioulasso, 01 Burkina Faso; IRD, UR 224 Maladies Infectieuses et Vecteurs: Ecologie, Génétique, Evolution et Contrôle (MIVEGEC), Montpellier, France; Département des Sciences et Techniques de l’Elevage (DSTE/FASE), Université Dan Dicko Dan Koulodo, BP 465, Maradi, Niger; CNRS, Université Montpellier, UMR 5290 MIVEGEC, Montpellier, France

**Keywords:** *Ehrlichia ruminantium*, Transovarial transmission, *Rhipicephalus microplus*, Heartwater

## Abstract

**Background:**

The invasion of West Africa by *Rhipicephalus microplus* during the past decade has changed the ecological situation of the agent of heartwater *Ehrlichia ruminantium* in this area. Before, its local vector, *Amblyomma variegatum,* was the most abundant tick species found on livestock. Today, the abundance of the *R. microplus* is one magnitude higher than that of *A. variegatum* in many west-African localities. We investigated the potential of this new ecological situation to impact the circulation of *E. ruminantium* in West Africa.

**Methods:**

*Ehrlichia ruminantium* infections were assessed with the specific PCR-diagnosis targeting the PCS20 region. This screening was applied on field samples of 24 *R. microplus* adults, on four females from a laboratory strain that had been blood-fed since larvae on one *E. ruminantium*-infected steer as well as on the offspring of these females at egg and larval stages.

**Results:**

The PCR detected *E. ruminantium* in 29 % of the field-collected *R. microplus*, i.e. twice as much as reported for *A. variegatum* with the same protocol. Regarding the laboratory strain, the PCR-diagnosis performed showed that all females were infected and passed the rickettsia to their progeny. Sequencing of the PCR product confirmed that the maternally inherited rickettsia was *E. ruminantium*.

**Conclusion:**

According to the present findings, the invasive dynamic of *R. microplus* in West Africa is currently impacting the local evolutionary conditions of *E. ruminantium* since it offers new transmission roads such as maternal transmission in *R. microplus*.

**Electronic supplementary material:**

The online version of this article (doi:10.1186/s13071-016-1651-x) contains supplementary material, which is available to authorized users.

## Background

*Ehrlichia ruminantium,* an obligatory intracellular rickettsia, is the causative agent of heartwater, a tick-borne disease that circulates throughout sub-Saharan Africa, the Caribbean and Indian Ocean islands [1]. Heartwater imposes a high economic cost to livestock industries since it induces high mortality (up to 80 %) in susceptible animals, especially goats and sheep [2]. *Ehrlichia ruminantium* is transmitted transstadially by three-host ticks of the genus *Amblyomma* with transovarial transmission reported only in *Amblyomma hebraeum* [3].

In West Africa, the only vector present, *Amblyomma variegatum*, was the most abundant tick-species encountered on livestock [4–6] until the accidental introduction of *Rhipicephalus microplus* in the early 2000s [7]. The newly introduced tick was so successful to invade this region that its abundance is currently a magnitude higher than that of *A. variegatum* in many west-African localities [8]. As a result, *R. microplus* is currently representing more than 60 % of the cattle tick-burden [8, 9] and is expected to frequently face *E. ruminantium*-infection risk in West Africa where the prevalence of *E. ruminantium* ranges from 39 to 61 % in cattle and from 28 to 51 % in sheep and goats [10, 11]. Noting that *E. ruminantium* was successfully cultured in *R. microplus* cell-lines [12] and that natural *E. ruminantium* infections of *R. microplus* were reported in the Caribbean [13], we investigated the ability of *E. ruminantium* to successfully infect the *R. microplus* ticks present in West Africa.

## Methods

We started the screening for *E. ruminantium* infections with a sample of 24 *R. microplus* adults collected in Benin (*n* = 7), Burkina Faso (*n* = 11) and Côte d’Ivoire (*n* = 6). We then detected *E. ruminantium* infection in one of three steers entering into the facilities of the International Center for Research-Development on livestock in Subhumid area (CIRDES, Bobo-Dioulasso, Burkina Faso). Freshly-hatched larvae of the Kimini strain (created by a sample of *R. microplus* collected on cattle at Kimini, Burkina Faso in July 2014) were allowed to complete their parasitic life-cycle on the *E. ruminantium*-infected steer. Five weeks later, four fully-engorged female ticks of the Kimini strain were allowed to lay eggs in individual vials before preserving them in 70 % ethanol until DNA extraction. The descent of each female was divided in two halves in order to be preserved in 70 % ethanol either as eggs or as freshly hatched (< 15 day-old) larvae.

Ticks were washed with PBS (phosphate buffered saline) buffer before proceeding to DNA extraction using the DNeasy Blood & Tissue Kit (QIAGEN, Hilden, Germany) according to the manufacturer’s instructions. *Ehrlichia ruminantium* infection was detected using the semi-nested PCR targeting the PCS20 genomic region [14]. A template of one field-collected *A. variegatum* specimen that had previously shown to be infected by *E. ruminantium* [4] was used as a positive control. PCR-products were purified and sent for sequencing (EUROFINS, Ebersberg, Germany). The newly-generated sequences were submitted in the GenBank database under accession numbers KX356089–KX356091. The phylogenetic relationships among the sequences generated in the present study and those of reference strains retrieved from GenBank (Additional file 1) were analysed with the Maximum Likelihood heuristic implemented in MEGA [15].

## Results and discussion

**Fig. 1:**
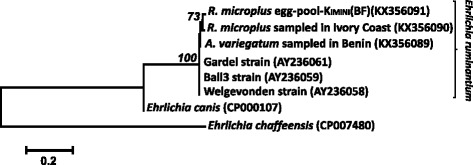
Phylogenetic relationships between the sequences generated in the present study and sequences for reference strains (GenBank accession numbers in parentheses) using maximum likelihood. A discrete Gamma distribution with invariant sites (*G* = 2.38 and *I* = 6.55 %) was used to model evolutionary rate differences among sites. Branch numbers indicate percent bootstrap support (5,000 bootstraps). The scale-bar is in units of substitutions/site. BF refers to Burkina Faso

The PCR-diagnosis showed the presence of *E. ruminantium* DNA in seven of the 24 field-collected *R. microplus* ticks (Table 1). The same result was obtained for each of the four females of the Kimini strain that had been fed on the infected steer (Table 1). These results could refer to the successful infection of *R. microplus* ticks by *E. ruminantium* and/or to the persistence of undigested DNA of the pathogen in tick blood-meals. This later hypothesis is however ruled out by the detection of *E. ruminantium* DNA in the descent of each laboratory female (Table 1). The female ticks exposed to *E. ruminantium* infection were all successfully infected and able to transmit the rickettsia to their offspring. Even if the specificity of the PCR-diagnosis method was already settled [16, 17], we confirmed our results by sequencing the PCR-products obtained from the field-collected *A. variegatum* positive control, one field-collected *R. microplus* adult from Ivory Coast and the descent of the kimini females. This provided sequences of high quality (in both forward and reverse directions) except in three egg-pools. The newly-generated sequences were shown to belong unambiguously to *E. ruminantium* (Fig. 1).

**Table 1:** Detection of *Ehrlichia ruminantium* in *Rhipicephalus microplus* ticks

Sample origin	Number of positive cases obtained via	
	PCR	PCS20 sequences
Field sampling		
Benin (*n* = 7)	3	na
Burkina Faso (*n* = 11)	2	na
Ivory Coast (*n* = 6)	2	1 obtained out of 1 attempt
KIMINI strain		
Engorged females (*n* = 4)	4	na
Egg pools (*n* = 4)	4	1 obtained out of 4 attempts
Larval pools (*n* = 4)	4	na

Therefore, in Kimini strain, the evaluation of *R. microplus* mothers that had passed *E. ruminantium* to their offspring would be 25 or 100 % according to the sequencing or PCR-diagnosis results, respectively. For the sake of comparison, most of the attempts to document transovarial transmission of *E. ruminantium* in *Amblyomma* spp. vectors failed with the exception of one study performed on *A. hebraeum* [3]. In the latter study, 40 *A. hebraeum* females previously exposed to *E. ruminantium* infection have been dispatched in five groups of eight females and the infection status of the progeny produced by each group has been evaluated through their ability to induce immunity and/or pathology in the susceptible sheep on which they blood-fed as larvae, nymphs or adults [3]. One of the five groups of offspring (20 %) was found to transmit heartwater as soon as the larval stage but three (60 %) induced pathology and/or promoted immunity as adults; such an increase from larval to adult stage has been interpreted as an increase of infectivity resulting from the multiplication of the pathogen in the tick-individuals [3].

The present results highlight that the recent changes in west-African tick communities resulting from *R. microplus* invasion [8, 9] is very likely to impact the circulation of *E. ruminantium* in West Africa, and thus the constraints modelling its evolution there. The high rate of circulation of *E. ruminantium* [10, 11, 20, 21] and the invasive dynamics of *R. microplus* in West Africa [8, 9, 18, 19] suggested a high rate of contact between these two species in the region. We presently confirmed this expectation by detecting *E. ruminantium* in 29 % field-collected *R. microplus* adults while only 10–16 % field-collected nymphs and adults *A variegatum* were reported infected by *E. ruminantium* with the same protocol [20, 21]. The possibility of high rate of maternal inheritance in *R. microplus*, that was demonstrated in the present study in the Kimini strain of Ivorian origin can thus drive some *E. ruminantium* genotypes to strictly adapt to this mode of transmission (i.e. to evolve toward a tick-endosymbiont life-cycle). Complementarily, as *R. microplus* is a one tick-species, the maternal-inheritance of *E. ruminantium* opens the possibility for this invasive species to play a role in heartwater epidemiology. Indeed, it was demonstrated that the repeated multiplication in *R. microplus* cells did not impact the infectivity of *E. ruminantium* for bovine endothelial cells [12]. Moreover, as many sheep breeds are highly sensitive to heartwater, it is noteworthy that *R. microplus* can feed on sheep in experimental settings [22] as well as in natural conditions in Burkina Faso [23]. All these data converge to support the hypothesis that the transovarial transmission of *E. ruminantium* in *R. microplus* might profoundly impact heartwater epidemiology in West Africa. To quantify such an impact, other parameters remain to be evaluated, such as the efficiency of *R. microplus* to transmit the maternally-inherited *E. ruminantium* to ruminants (mainly sheep and goats) or the possible variation in *E. ruminantium* virulence between this potential vector and the known tick-vector.

## Additional file

Additional file 1: Alignment of the sequences generated in the present study with those of *Ehrlichia* spp. references strains retrieved from the GenBank database. (TXT 2 kb)
